# Comparative evaluation of screening tools for sarcopenia in patients with axial spondyloarthritis

**DOI:** 10.1038/s41598-024-65120-2

**Published:** 2024-06-22

**Authors:** Sumapa Chaiamnuay, Natnicha Kanjanavaikoon, Pannarat Saisirivechakun

**Affiliations:** 1 Rheumatic Disease Unit, Department of Internal Medicine, Phramongkutklao Hospital and Phramongkutklao College of Medicine, 315 Ratchawithi Road Ratchathewi District, Bangkok, 10400 Thailand; 2 Department of Internal Medicine, Krathumbaen Hospital, Samut Sakhon, Thailand; 3 Department of Internal Medicine, Nakhon Pathom Hospital, Nakhon Pathom, Thailand

**Keywords:** Early detection Screening, Screening, Axial spondyloarthritis, Sensitivity and specificity, Sarcopenia, Muscle wasting, Diseases, Rheumatology

## Abstract

Sarcopenia is linked to chronic inflammation and muscle wasting. This research aims to compare the screening accuracy of tools for sarcopenia in axial spondyloarthritis (axSpA). A cross-sectional study involving 104 axSpA patients was conducted at Phramongkutklao Hospital between January 2020 and February 2021. Sarcopenia was diagnosed according to the AWGS 2019 criteria. Appendicular skeletal muscle mass was measured using DXA. SARC-F, SARC-CalF, and SARC-F+EBM, muscle strength, and physical performance were assessed. The screening tests were evaluated using ROC curves. The optimal cutoffs were identified with the Youden index. Most patients were male (74%), with a mean (SD) age and disease duration of 42.6 (12.22) and 8.3 (8.5), respectively. The prevalence of sarcopenia was 22.1%. The AUCs (95% CI) for calf circumference, SARC-F, SARC-CalF, SARC-F+EBM, handgrip strength, chair stand time, gait speed, and time and go test were 0.830 (0.734, 0.925), 0.509 (0.373–0.645), 0.782 (0.670–0.894), 0.856 (0.758–0.954), 0.710 (0.594–0.825), 0.640 (0.508–0.772), 0.689 (0.539–0.839), and 0.711 (0.576–0.846), respectively. The optimal cutoffs for SARC-F, SARC-CalF, and SARC-F+EBM were 1, 10, and 10, with sensitivity/specificity of 81.0%/29.7%, 90.5%/68.9%, and 77.3%/87.2%, respectively. Calf circumference, SARC-CalF, and SARC-F+EBM had the best performance to screen for sarcopenia in axSpA patients. Lowering the thresholds would potentially enhance the performances of SARC-CalF and SARC-F+EBM.

## Introduction

Axial spondyloarthritis (axSpA) is a chronic inflammatory condition primarily affecting the axial joints. Inflammatory cytokines such as interleukin-1 (IL-1), IL-6, IL-17/23, and tumor necrosis factor-alpha prompt the production of receptor activator of nuclear factor kappa-B ligand, a potential catalyst for osteoclast differentiation, leading to bone loss in individuals with ax-SpA. Concurrently, bone morphogenetic protein and Wnt signaling pathways encourage osteoblast differentiation, contributing to the formation of new bone and eventually resulting in ankylosis and disability^1^.

Sarcopenia, as per the 2010 criteria established by the European Working Group on Sarcopenia in Older People (EWGSOP), is characterized by abnormally low muscle mass along with reduced skeletal muscle strength and/or impaired physical performance. It is associated with physical disability, decreased quality of life, increased hospitalization, and mortality^2^. Primary sarcopenia initially described the decline in muscle mass, function, and strength associated with aging. Nevertheless, secondary sarcopenia has been identified, characterized by expedited muscle mass reduction in various conditions, including inflammatory diseases, nutritional deficiencies, and limitations in physical activity. The Asian Working Group for Sarcopenia (AWGS) published a diagnostic algorithm in 2014, based on Asian data^3^, which consists of three domains, similar to the EWGSOP criteria. This algorithm was later updated in 2019^4^.

Sarcopenia predominantly affects the elderly; however, younger individuals with chronic diseases are also at risk. The National Health and Nutrition Examination Survey (NHANES) database highlighted that increased systemic immune inflammation escalates the risk of sarcopenia among younger individuals aged 18–59^5^. Therefore, axSpA patients have an elevated risk of developing sarcopenia due to the substantial burden of systemic inflammation. Additionally, these inflammatory cytokines contribute to increased muscle catabolism, prompting muscle loss, and down-regulation of anabolic hormones. Moreover, chronic inflammation stemming from ax-SpA can lead to chronic musculoskeletal pain, subsequently reducing physical activities and diminishing muscle utility. The prevalence of sarcopenia in axSpA was reported between 15 and 62% depending on the patients’ characteristics, the diagnostic approaches and criteria, and the methods of skeletal muscle measurements^6^. Sarcopenia is associated with high disease activity, poor functioning, and low bone mineral density in axSpA patients^6^.

Recognizing and treating sarcopenia is important because of the associated adverse health outcomes, including disability, insulin resistance, falls, frailty, and mortality. Given the high prevalence of sarcopenia in axSpA patients, despite their relatively young age and the adverse health outcomes, screening and early diagnosis of sarcopenia are important. Our study is aimed at identifying the most appropriate screening tools for sarcopenia in axSpA patients.

## Method

Between January 2020 and February 2021, a cross-sectional study involving 104 patients was conducted at Phramongkutklao Hospital in Bangkok, Thailand. The study's inclusion criteria required consecutive ax-SpA patients who met the 2009 Assessment of Spondyloarthritis International Society (ASAS) criteria, were at least 18 years old, and were willing to participate in the study. The exclusion criteria included having active cancer, pregnancy, breastfeeding, high-dose steroid use, an inability to undergo a physical test, and limitations regarding dual-energy X-ray absorptiometry (DXA), such as recent exposure to barium or radionuclides within the past 2 weeks. The study received approval from the Royal Thai Army Institutional Board Review under the reference R011h/63, and written informed consent was obtained from all participants prior to their participation.

### Screening tools for sarcopenia

This study aimed to investigate the following screening tools for sarcopenia in patients with axial spondyloarthritis (axSpA).

1. The SARC-F is composed of five screening questions that assess muscle strength, walking ability, rising from a chair, climbing stairs, and the self-reported number of falls in the previous year. Each question can be answered on a scale from 0 to 2, resulting in a total score ranging from 0 to 10. A score equal to or greater than 4 is considered the cutoff for a positive screening test for sarcopenia^7^.
2. SARC-CalF is comprised of the SARC-F questionnaire along with an additional 10 points assigned for having a maximum calf circumference equal to or less than 34 cm for men and 33 cm for women. The SARC-CalF score has a range of 0 to 20, and a score equal to or greater than 11 is considered the cutoff for a positive screening test for sarcopenia^8^.
3. SARCF + EBM is composed of the SARC-F questionnaire and incorporates scores based on age and BMI assessments. For age, patients aged < 75 years old were assigned a score of 0, while patients ≥ 75 years old received a score of 10. Regarding BMI, patients with a BMI > 21 kg/m^2^ were assigned a score of 0, whereas those with a BMI ≤ 21 kg/m^2^ received a score of 10. The SARC-F + EBM score has a range of 0–30, and a score equal to or greater than 12 is utilized as the cutoff for a positive screening test for sarcopenia^9^.
4. Handgrip strength assessment was assessed by a digital hand dynamometer set at the fifth strength level. Initially, participants used their dominant hand while seated, maintaining a 90-degree elbow flexion, and performed three grip tests. The best performance from these tests was recorded in kilograms. The thresholds specified in the AWGS 2019 guidelines for categorizing low handgrip muscle strength stand at < 28.0 kg for men and < 18.0 kg for women^4^. These cutoff values were derived from normative data gathered from a combined dataset of eight cohorts in Asia^10^.
5. The chair stand time records the time needed to complete five repetitions of standing up and sitting down in a chair, with the measurement recorded in seconds. A completion time of 12 s or more is considered the cutoff for low physical performance^4^.
6. The gait speed test measures the time required for participants to walk 6 m at a standard pace, with two trials conducted, and then calculates the resulting average in meters per second. A gait speed of less than 1.0 m per second in the 6-m walk is considered the cutoff for low physical performance^4^.
7. The time up-and-go test measures the time from when participants initially sit in the chair to the moment they stand up, walk three meters forward at their usual walking speed, turn around at a designated marker, walk back to the chair, and sit down again. The time up-and-go test of more than 10 s is considered the cutoff for low physical performance^11^. However, the time up-and-go test was not included in the diagnosis of sarcopenia by the AWGS 2019 criteria.

### Definition of sarcopenia

Sarcopenia was diagnosed following the AWGS 2019 clinical research setting pathway algorithm^4^. Muscle strength and physical performance were assessed by the same investigation (NK). Appendicular skeletal muscle mass (ASM) was measured by anteroposterior dual-energy X-ray absorptiometry (DXA), using the GE-Lunar iDXA (#210754) and GE-Lunar DPX Duo densitometer (GE Healthcare, Madison, WI, USA). The lean mass of the arms and legs was considered equivalent to the skeletal muscle mass. To determine the skeletal muscle mass index (SMI), the lean mass was divided by height squared (kg/m^2^). The appendicular SMI was calculated as the sum of arm and leg SMIs (kg/m^2^). According to the AWGS 2019 criteria for diagnosing sarcopenia, individuals are categorized as having low muscle mass if their SMI falls below 7.0 kg/m^2^ for men and 5.4 kg/m^2^ for women^4^.

In accordance with the AWGS 2019 recommendations, 'possible sarcopenia' is defined as decreased muscle strength assessed through hand grip strength, either independently or in conjunction with reduced physical performance evaluated using chair stand time and gait speed tests. In contrast, 'sarcopenia' is characterized by low ASM and either reduced muscle strength or impaired physical performance, whereas “severe sarcopenia” is defined as low ASM, low muscle strength, and low physical performance^4^.

### Statistically analyses

Variables with a normal distribution were presented as the mean and standard deviation (SD), and non-normal quantitative variables were presented as the median and interquartile range (IQR). Sensitivity, specificity, positive likelihood ratio (+ LR), negative likelihood ratio (− LR), positive predictive value (PPV), and negative predictive value (NPV) were computed by using the AWGS 2019 criteria as the gold standard for the diagnosis of sarcopenia. The diagnostic accuracy of the SARC-F, SARC-CalF, SAR-F+EBM, hand grip strength, chair stand time, gait speed, and the time up-and-go test were calculated using receiver operating characteristic (ROC) curves, and a 95% confidence interval (CI). The Youden index (Sensitivity + Specificity − 1) was used to determine the ROC data cutoff point. The differences in sensitivity, specificity, PPV, and NPV between the screening tests were investigated by the Chi-square test with the Marascuillo procedure. Comparisons between ROC curves were examined using the DeLong method. Statistical analyses were performed using SPSS software (IBM SPSS Statistics for Windows, Version 23.0. Armonk, NY: IBM Corp. All statistical tests were 2-sided. A p-value of less than 0.05 was considered statistically significant.

### Ethics approval

The study received approval from the Royal Thai Army Institutional Board Review under the reference R011h/63. This study has been performed in accordance with the Declaration of Helsinki. Written informed consent was obtained from all participants prior to their participation.

## Result

This study comprised 104 ax-SpA patients, with the majority being male (74%). The patients were presented with a mean (SD) age and disease duration of 42.6 (12.2) and 8.3 (8.5) years, respectively. The prevalence of sarcopenia, severe sarcopenia, and possible sarcopenia in axSpA patients were 22.1% (23/104), 4.8% (5/104), and 85.6% (89/104), respectively. There were two patients who could not perform a chair stand time test, and one patient could not perform a time up and go test because of severe low back pain.

Extra-articular manifestations, such as anterior uveitis and psoriasis, were observed in 5.8% and 1.5% of the ax-SpA patients, respectively. Regarding current treatment, non-steroidal anti-inflammatory drugs (NSAIDs) were prescribed to 58 patients (55.8%), glucocorticoids to 7 patients (6.7%), and conventional synthetic disease-modifying antirheumatic drugs (csDMARDs) to 78 patients (75%). Methotrexate and sulfasalazine were the most commonly prescribed csDMARDs. Additionally, 26 patients (25%) were receiving biologic agents, with anti-tumor necrosis factor (anti-TNF) agents being the most frequently used (23 patients), while only three patients were receiving anti-interleukin-17 (anti-IL-17) agents.

The mean Ankylosing Spondylitis Disease Activity Score (ASDAS) was 2.6 (SD = 1.2), with 40 patients having high disease activity and 26 patients having very high disease activity. The mean Bath Ankylosing Spondylitis Functional Index (BASFI) score, which assesses functional impairment, was 2.4 (SD = 2.4). Significant functional limitations (BASFI > 4) were found in 20 patients (19%). The details of patients’ characteristics and the differences between axSpA patients with and without sarcopenia were described in the previous report^6^.

Among axSpA patients, individuals with sarcopenia exhibited notably higher scores in screening tools such as SARC-CalF and SARCF+EBM, along with reduced calf circumference in comparison to those without sarcopenia (median 12 vs. 3, p < 0.001; 11 vs. 3, p < 0.001; mean (SD) 31.4 (3.7) vs. 35.6 (3.7), p < 0.001, respectively). However, no significant difference emerged in SARC-F scores between the two groups (median 2 vs. 2, p = 0.693). AxSpA patients with sarcopenia displayed lower mean (SD) muscle strength, as measured by handgrip strength, than those without sarcopenia (27.1 (10.6) vs. 35.6 (10.4), p = 0.001). Regarding muscle performance, individuals with sarcopenia took longer to complete the chair stand time and time up and go test, showing means (SD) of 18.4 (7.4) vs. 15.3 (6.6), p = 0.067, and 12.2 (3.4) vs. 10.3 (2.8), p = 0.009, respectively. Conversely, there was no discrepancy in gait speed between individuals with and without sarcopenia, with medians of 1.2 vs. 1.0, p = 0.419. Details regarding sarcopenia assessment in axSpA patients are outlined in Table 1.

**Table 1:** Sarcopenia assessments of axial spondyloarthritis patients with and without sarcopenia.

	Non-sarcopenia (n = 81)	Sarcopenia (n = 23)	p-value
SARC-F, median (min, max)	2 (0, 10)	2 (0, 9)	0.902^a^
High SARC-F score, n (%)	18 (24.3)	6 (28.6)	0.693^b^
Calf circumference (cm), mean (SD)	35.6 (3.7)	31.4 (2.7)	< 0.001^d^
SARC-CalF score, median (min, max)	3 (0, 20)	12 (0, 19)	0.0001^a^
High SARC-CalF score, n (%)	16 (21.6)	16 (76.2)	< 0.001^b^
SARCF + EBM, median (min, max)	3 (0, 14)	11 (1, 19)	< 0.001^a^
High SARCF + EBM, n (%)	3 (3.9)	9 (40.9)	< 0.001^c^
Skeletal muscle mass index (kg/m^2^), mean (SD)	7.6 (1.3)	5.8 (0.9)	< 0.001^d^
Low skeletal muscle mass index, n (%)	5 (6.2)	23 (100)	< 0.001^b^
Hand grip strength (kg), mean (SD)	35.6 (10.4)	27.1 (10.6)	0.001^d^
Low grip strength, n (%)	5 (6.2)	7 (30.4)	0.001^b^
Chair stand time* (s), mean (SD)	15.3 (6.6)	18.4 (7.4)	0.067^d^
Increased chair stand time, n (%)	54 (66.7)	20 (87.0)	0.001^b^
Gait speed (m/s), median (min, max)	1.0 (0.6, 10.6)	1.2 (0.7, 4.1)	0.419^a^
Low gait speed, n (%)	32 (39.5)	5 (21.7)	0.116^b^
Time up and go** (s), mean (SD)	10.3 (2.8)	12.2 (3.4)	0.009^d^
High time up and go, n (%)	2 (2.5)	2 (8.7)	0.211^c^

^a^Two-sample Wilcoxon rank-sum test.

^b^Pearson chi-square test.

^c^Exact probability test.

^d^Independent t-test.

Table 2 demonstrates the characteristics of the screening tools including sensitivity, specificity, positive and negative likelihood ratios, positive and negative predictive values, and Receiver Operating Curve (ROC) models for identifying sarcopenia as defined by AWGS 2019 criteria as the reference standard. The three most sensitive tools for detecting sarcopenia in AxSpA patients were chair stand time (87.0%, 95% CI 66.4–97.2), calf circumference (85.7%, 95% CI 63.7–97.0), and SARC-CalF (76.2%, 95% CI 52.8–91.8). Conversely, the three least sensitive tools were TUG (8.7%, 95% CI 1.1–28.0), gait speed (21.7, 95% CI 7.5–43.7) and SARC-F (28.6%, 95% CI 11.3–52.2). The three most specific tools for detecting sarcopenia in AxSpA patients were TUG (97.5%, 95% CI 91.4–99.7), SARCF + EBM (96.2%, 95% CI 89.2–99.2), and handgrip strength (93.8%, 95% CI 86.2–98.0).

**Table 2:** Screening tests’ characteristics of calf circumference, SARC-F, SARC-CalF, SARCF + EBM, handgrip strength, chair stand time, time up-and-go, and gait speed against the AWGS 2019 criteria.

Screening test	Sensitivity% (95% CI)^a^	Specificity (95% CI)^a^	Positive likelihood ratio (95% CI)	Negative likelihood ratio (95% CI)	Positive predictive value^a^ % (95% CI)	Negative predictive value % (95% CI)	Area under the curve^B^ (95%CI)
1. Calf circumference	85.7 (63.7, 97.0)^2,5,7,8^	77.0 (65.8, 86.0)^6,7^	3.73 (2.37, 5.86)	0.19 (0.06, 0.53)	51.4 (34, 68.6)	95.0 (86.1, 99.0)	0.830 (0.734, 0.925)
2. SARC-F	28.6 (11.3, 52.2)^1,6^	75.7 (64.3, 84.9)^4,6,7^	1.17 (0.53, 2.58)	0.94 (0.7, 1.27)	25 (9.8, 46.7)	78.9 (67.6, 87.7)	0.509 (0.373, 0.644)^b^
3. SARC-CalF	76.2 (52.8, 91.8)^7,8^	78.4 (67.3, 87.1)^6,7^	3.52 (2.15, 5.78)	0.3 (0.14, 0.66)	50 (31.9, 68.1)	92.1 (82.4, 97.4)	0.782 (0.669, 0.895)
4. SARCF + EBM	40.9 (20.7, 63.6)	96.2 (89.2, 99.2)^2,6,8^	10.64 (3.15, 35.95)	0.61 (0.43, 0.87)	75 (42.8, 94.5)^8^	85.2 (76.1, 91.9)	0.860 (0.764, 0.955)
5. Handgrip strength	30.4 (13.2, 52.9)^1.6^	93.8 (86.2, 98.0)^6,8^	4.93 (1.73, 14.09)	0.74 (0.56, 0.98)	58.3 (27.7, 84.8)	82.6 (73.3, 89.7)	0.710 (0.593, 0.826)^b^
6. Chair stand time	87.0 (66.4, 97.2)^2,5,7,8^	33.3 (23.2, 44.7)^1,2,3,4,5,7^	1.30 (1.05, 1.63)	0.39 (0.13, 1.17)	27.0 (17.4, 38.6)	90.0 (73.5, 97.9)	0.669 (0.545, 0.794)^b^
7. Time up and go	8.7 (1.1, 28.0)^1,3,6^	97.5 (91.4, 99.7)^1,2,3,6,8^	3.52 (0.52, 23.65)	0.94 (0.82, 1.07)	50.0 (6.8, 93.2)	79.0 (69.7, 86.5)	0.712 (0.585, 0.839)
8. Gait speed 6 m walk	21.7 (7.5, 43.7)^1,3,6^	60.5 (49.0, 71.2)^4,5,7^	0.55 (0.24, 1.25)	1.29 (0.98, 1.71)	13.5 (4.5, 28.8)^4^	73.1 (60.9, 83.2)	0.687 (0.545, 0.829)^b^

^a^p-value < 0.05 by chi-square test among Calf circumference, SARC-F, SARC-CalF, SARCF + EBM, Handgrip strength, Chair stand time, Time up and go, and Gait speed 6 m walk.

^1^^–^^8^p-value < 0.05 for multiple proportions test by Marascuillo procedure.

^1^Significantly different with Calf circumference (p < 0.05).

^2^Significantly different with SARC-F (p < 0.05).

^3^Significantly different with SARC-CalF (p < 0.05).

^4^Significantly different with SARCF + EBM (p < 0.05).

^5^Significantly different with Handgrip strength (p < 0.05).

^6^Significantly different with Chair stand time (p < 0.05).

^7^Significantly different with Time up and go (p < 0.05).

^8^Significantly different with Gait speed 6 m walk (p < 0.05).

^B^p-value < 0.05 by the DeLong method.

^b^Significantly different with Calf circumference (p < 0.05).

**Figure 1:**
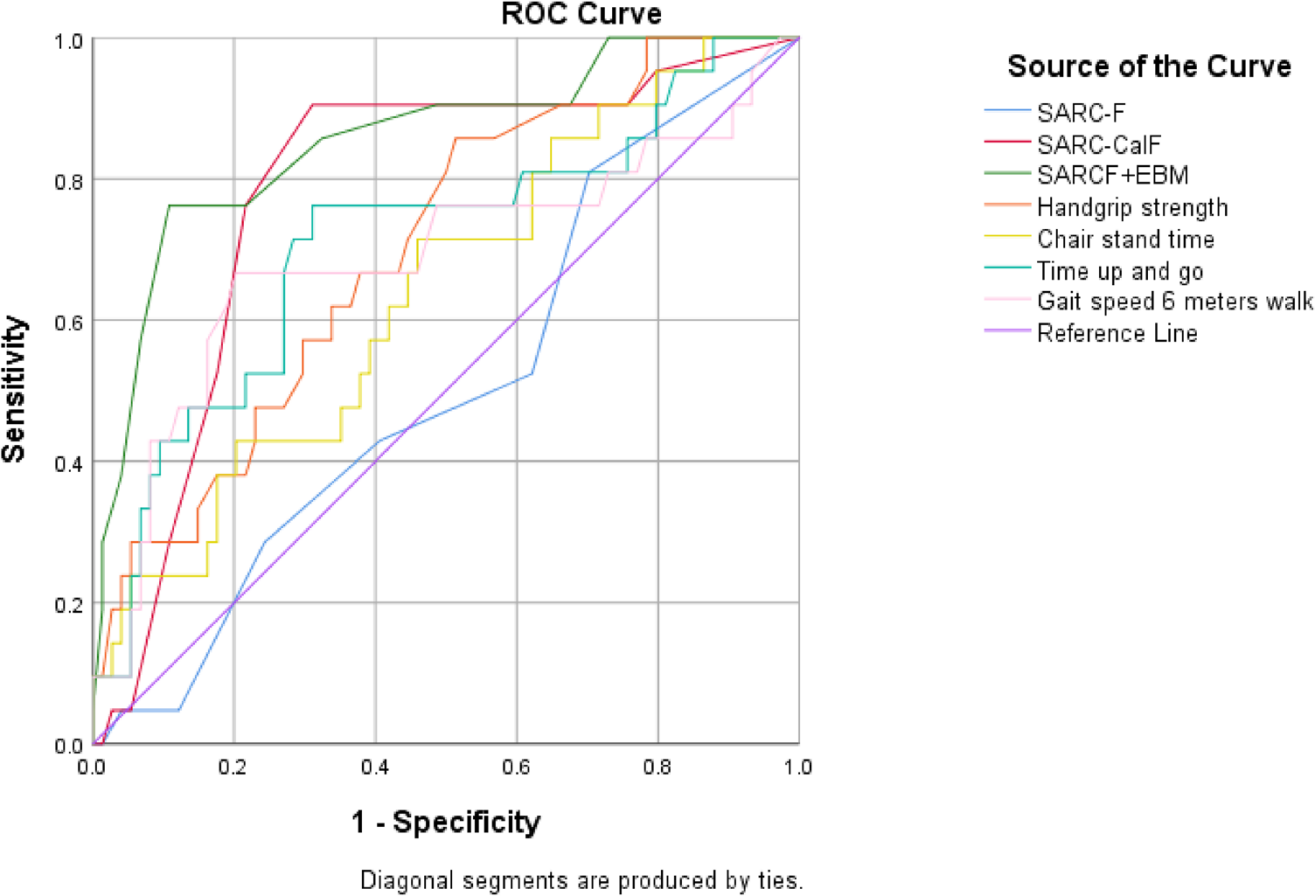
Receiver operating characteristic (ROC) curve analysis of sarcopenia screening tests in relation to sarcopenia defined according to AWGS 2019 criteria in axial spondyloarthritis patients.

SARCF + EBM exhibited the highest positive likelihood ratio of 10.64 (95% CI 3.15–35.95), the highest PPV of 75% (95% CI 42.8–94.5), and the highest AUC of 0.860 (95% CI 0.764–0.955). Both SARCF + EBM and calf circumference showed good overall accuracy, with AUCs (> 0.8) in screening for sarcopenia in AxSpA patients. In contrast, SARC-F demonstrated poorer performance compared to calf circumference, SARC-CalF, and SARC-F + EBM, with AUCs (95% CI) of 0.509 (0.373–0.644), 0.830 (0.734–0.925), 0.782 (0.669, 0.895), and 0.860 (0.764, 0.955), respectively. The ROC curves illustrating screening tests against the AWGS 2019 criteria for sarcopenia are presented in Fig. 1.

We further investigated the optimal cutoffs for sarcopenia screening in AxSpA patients using SARC-CalF and SARCF + EBM based on the Youden index. The optimal SARC-CalF threshold associated with sarcopenia was determined as 10, with 90.5% sensitivity, 68.9% specificity, and a 45.2% false positive rate. Comparing this threshold (SARC-CalF ≥ 10) with the one used in general populations (SARC-CalF ≥ 11), SARC-CalF ≥ 11 showed reduced sensitivity of 76.2% but increased specificity of 78.2%. Likewise, for SARCF + EBM, the best Youden index threshold was 10, presenting 77.3a% sensitivity, 87.2% specificity, and a 63% false positive rate, contrasting with the threshold for general population screening (SARC-CalF ≥ 12), which exhibited only 40.9% sensitivity, 96.2% specificity, and a 75% false positive rate. Table 3 displays the performance characteristics of SARCF, SARCF + EBM, and SARC-CalF, including sensitivity, specificity, false positive rate, and the Youden index.

**Table 3:** Performance characteristics at each cutoff of SARC-F (a), SARC-CalF (b), and SARC-F + EBM (c) with sensitivity, specificity, false positive rate, and Youden index.

Test	Cutoff ≥	Sensitivity	Specificity	False positive rate	Youden index
a. SARC-F ≥					
	1	81.0	29.7	24.6	0.107
	2	52.4	37.8	19.3	− 0.098
	3	42.9	59.5	23.1	0.024
	4	28.6	75.7	25.0	0.043
	5	4.8	87.8	10.0	− 0.074
b. SARC-CalF ≥					
	8	90.5	67.6	44.2	0.570
	9	90.5	67.6	44.2	0.570
	10	90.5	68.9	45.2	0.584
	11	76.2	78.4	50.0	0.536
	12	52.4	82.4	45.8	0.338
c. SARCF + EBM ≥					
	8	77.3	84.6	58.6	0.609
	9	77.3	84.6	58.6	0.609
	10	77.3	87.2	63.0	0.635
	11	59.1	93.6	72.2	0.517
	12	40.9	96.2	75.0	0.361

Youden index = (Sensitivity + Specificity − 1).

## Discussion

The prevalence of sarcopenia is considerable, affecting approximately one-fifth (22.1%) of axSpA patients. Furthermore, sarcopenia was reported to be associated with poor function as measured by BASFI score^6^, underscoring the importance of screening for sarcopenia in axSpA patients. The SARC-F questionnaire is straightforward and stands as a convenient tool for routine use in rheumatology clinics. With good reliability, it has been translated into numerous languages as well as validated for the uses in various diseases and patient populations^12^. It is recommended by EWG-SOP2 and AWGS 2019 recommendations as the screening tool in the sarcopenia case-finding strategy. However, the present study reveals that SARC-F has low sensitivity (28.6%) and low diagnostic accuracy (AUC 0.509) in identifying sarcopenia among axSpA patients. These findings are aligned with prior studies conducted across various populations, including systemic sclerosis^13^, chronic kidney disease patients^14^, and the elderly^15^.

The characteristics of a good screening test depend on the prevalence of the disease. In the case of diseases with low prevalence, the screening test should exhibit high specificity and PPV. Conversely, for more common diseases, the screening test should prioritize high sensitivity and NPV. Given the common occurrence of sarcopenia, an ideal screening modality should emphasize high sensitivity and NPV. Consequently, SARC-F might not be an optimal tool as a screening test for identifying sarcopenia in axSpA patients. To enhance the sensitivity of SARC-F, several strategies have been proposed. First, the cutoff value of SARC-F can be adjusted. Lowering the cutoff of the SARC-F score to ≥ 1 increases the sensitivity and AUC^16^ with acceptable specificity^17,18^.

The second strategy involves integrating SARC-F with other variables such as calf circumference, age, or BMI. The present studies revealed that SARC-CalF and SARCF + EBM significantly improved sensitivity and diagnostic accuracy as compared to SARC-F alone. Consistent with our findings, prior studies reported that SARC-CalF significantly improves the sensitivity and overall diagnostic accuracy of SARC-F for screening sarcopenia in community-dwelling older adults as well as nursing home residents^19^, hemodialysis patients^20^, diabetes mellitus patients^21^, and advanced cancer patients^22^, regardless of which diagnostic criteria were used (EWGSOP2, AWGS 2019, International Working Group on Sarcopenia, and Foundation for the National Institutes of Health). Additionally, in patients experiencing chronic musculoskeletal pain, both SARC-F and SARC-CalF exhibited significant correlations with pain-related disability. Both tools proved reliable for screening sarcopenia in chronic musculoskeletal pain; however, SARC-CalF demonstrated higher reliability than SARC-F^23^.

SARC-F + EBM was initially proposed and validated by Kurita et al. in 2019 against AWGS and EWGSOP2 criteria for sarcopenia in 959 adult patients with musculoskeletal diseases scheduled for spinal surgery, knee, or hip replacement therapy^9^. Given that axSpA patients often present with hip and/or knee arthritis and chronic low back pain, resembling the cohort from the original SARC-F + EBM study, it is intriguing to assess its potential as a screening tool in axSpA. Our findings indicated a significant improvement in the sensitivity and overall diagnostic accuracy of SARC-F + EBM over SARC-F alone for sarcopenia screening. However, the sensitivity of SARC-F + EBM in this study was 40.9%, contrasting with the study by Kurita et al., which reported a higher sensitivity of 77.8%. This discrepancy in sensitivity could be attributed to the age difference among study participants, as the additional 10 score of SARC-F + EBM is assigned for ages ≥ 75 years old. Notably, axSpA patients in our study were younger compared to those with degenerative diseases in the other study, suggesting the necessity to consider a lower cutoff for SARC-F + EBM in axSpA patients, potentially below 12. Moreover, it's crucial to note the difference in the criteria used for diagnosing sarcopenia; our study employed AWGS2 criteria, while Kurita et al. used AWGS. Given the recent introduction of SARC-F + EBM, its validation has been limited to a few studies and specific patient populations.

The third strategy is to use calf circumference independently as an alternative to SARC-F and its modified versions for sarcopenia screening. This study demonstrated that the calf circumference exhibits high sensitivity, negative predictive value, and diagnostic accuracy as indicated by the AUC. Hence, it could serve as an effective screening tool for sarcopenia in axSpA patients. Low calf circumference has been reported to be correlated with low skeletal muscle mass, low muscle strength^24^, low physical performance^24^, sarcopenia^25^, and increased mortality^25^ in different settings (community, hospital, and nursing home settings) and various patient populations (parkinsonism, stroke, chronic liver diseases, and interstitial lung diseases)^26^. Despite these advantages, limitations exist, notably the variability in calf circumference measurements and the absence of standardized cutoff values tailored for diverse patient demographics such as age or ethnicity. Nevertheless, the AWGS 2019 recommends measuring calf circumference alone as one of the tools for the initial screening of sarcopenia^4^.

The diagnostic performance of the screening tools is reliant upon the chosen cutoff value. The cut-off SARC-CalF was first investigated in community-dwelling elderly aged 60 years or older in Brazil with a prevalence of sarcopenia of 8.3%. The best cutoff for sarcopenia is over 11, which results in the Youden index of 0.5 and the AUC of 0.736^8^. Conversely, in a study among community-dwelling older adults aged 75 years and older in Japan, where sarcopenia prevalence was 8.3%, the identified best cutoff for sarcopenia was over 7, exhibiting high sensitivity (94.7%), specificity (92.3%), and an AUC of 0.98^27^. In the present study, featuring a prevalence of sarcopenia of 22.1%, the optimal SARC-CalF cutoff with the highest Youden index was 10. Variations in cutoff values could be attributed to differences in patient populations, particularly age and sarcopenia prevalence, where lower prevalence necessitates higher cutoff scores. The best cutoff score for SARC-F + EBM from the present study is 10, which is lower than the cutoff from the original study at 12^9^, and the difference could likely be due to the younger age of axSpA patients in this study.

This study has several limitations. Firstly, it features a relatively small sample size, focusing exclusively on Thai or Asian participants. Therefore, the findings of this study need validation through larger-scale studies involving diverse ethnicities. Secondly, unlike rheumatoid arthritis, the implications of sarcopenia in axSpA have not been well established. Thirdly, universal sarcopenia screening is currently not recommended due to the absence of an identified or validated appropriate test. Furthermore, it remains to be demonstrated whether screening would yield health benefits that outweigh the associated costs.

## Conclusions

To the best of our knowledge, this study is the first to evaluate various tools for sarcopenia screening in axSpA. We found that calf circumference, SARC-CalF, and SARC-F + EBM had the best performance for identifying sarcopenia in axSpA patients. Our findings align with the AWGS 2019 guideline, endorsing sarcopenia case-finding strategies through the evaluation of calf circumference, SARC-F, or SARC-CalF, supplemented by SARC-F + EBM. In contrast, EWG-SOP2 recommends solely SARC-F, or clinical suspicion, for screening in the case-finding strategy. Hence, the AWGS 2019 approach appears more suitable for Asian axSpA patients due to the wider array of screening tools available during the case-finding process.

## Data Availability

The datasets are available from the corresponding author upon reasonable request.
